# Theoretical and Experimental Considerations for Investigating Multicomponent Diffusion in Hydrated, Dense Polymer Membranes

**DOI:** 10.3390/membranes12100942

**Published:** 2022-09-27

**Authors:** Antara Mazumder, Breanna M. Dobyns, Michael P. Howard, Bryan S. Beckingham

**Affiliations:** 1 Department of Chemical Engineering, Auburn University, Auburn, AL 36849, USA; 2 Department of Chemistry, University of South Alabama, Mobile, AL 36688, USA

**Keywords:** multicomponent diffusion, polymer membranes, dense films

## Abstract

In many applications of hydrated, dense polymer membranes—including fuel cells, desalination, molecular separations, electrolyzers, and solar fuels devices—the membrane is challenged with aqueous streams that contain multiple solutes. The presence of multiple solutes presents a complex process because each solute can have different interactions with the polymer membrane and with other solutes, which collectively determine the transport behavior and separation performance that is observed. It is critical to understand the theoretical framework behind and experimental considerations for understanding how the presence of multiple solutes impacts diffusion, and thereby, the design of membranes. Here, we review models for multicomponent diffusion in the context of the solution-diffusion framework and the associated experiments for characterizing multicomponent transport using diffusion cells. Notably, multicomponent effects are typically not considered when discussing or investigating transport in dense, hydrated polymer membranes, however recent research has shown that these effects can be large and important for understanding the transport behavior.

## 1. Introduction

Polymer membranes are generally thin polymeric films that allow the transport of certain species while limiting/inhibiting the transport of others. From dense to porous, amorphous to crystalline, hydrophobic to hydrophilic, synthetic polymeric membranes can be modified for a range of solutes and applications ranging from biomedical applications like kidney dialysis [1], energy applications like fuel cells and batteries [2,3], and water purification applications [4,5] such as ultra- and nano-filtration. Broadly, polymer membranes can be classified into two categories based on structure and transport mechanism: (1) non-porous or dense membranes, which typically have a homogeneous structure where transport occurs primarily through the dynamically evolving regions between polymer chains and (2) porous membranes, which have fixed pores that mediate transport. In this review, we focus our attention on approaches for modeling and experimentally investigating multicomponent transport phenomena in dense polymer membranes.

Dense polymer membranes are utilized in numerous applications to control the transport of molecules and/or ions in order to facilitate separation (e.g., gas separations [6,7], reverse osmosis, or desalination [8,9,10,11]) or, in the case of electrochemical devices (electrolyzers, direct methanol fuel cells, redox flow batteries, solar fuels devices [12], etc.), device cell operation as well as maintaining high device efficiencies. In many of these applications, the polymer membrane is challenged with complex mixtures of solutes. For instance, in gas separation applications, by necessity the polymer membrane must handle a mixture of gases, e.g., CO_2_ capture, where O_2_, N_2_, CO_2_ and other species may be present. In water desalination applications, multiple different salts (NaCl, CaCl, MgCl) are present, while in electrochemical applications, different mixtures of electrolyte as well as feed and product molecules/ions may be present in the device. In this review, we narrow our focus to hydrated, dense polymer membranes and highlight considerations for experimentally investigating solution diffusion transport for multicomponent systems.

Dense polymer membranes do not have fixed pores, but polymer chains cannot occupy the same volume and do not pack very efficiently, creating dynamic fractional free volume (space not occupied by polymer chains). The transport of molecules and/or ions occurs through this free volume and is commonly described using the solution-diffusion model (Section 2.1). In this model, the solute flux for a given concentration difference across the membrane is proportional to a permeability, *P_i_*, that is defined as the product of the kinetic diffusivity, *D_i_*, and the thermodynamic solubility, *K_i_*:

(1) $$P_{i}=D_{i}K_{i}\;.$$

**Figure 1:**
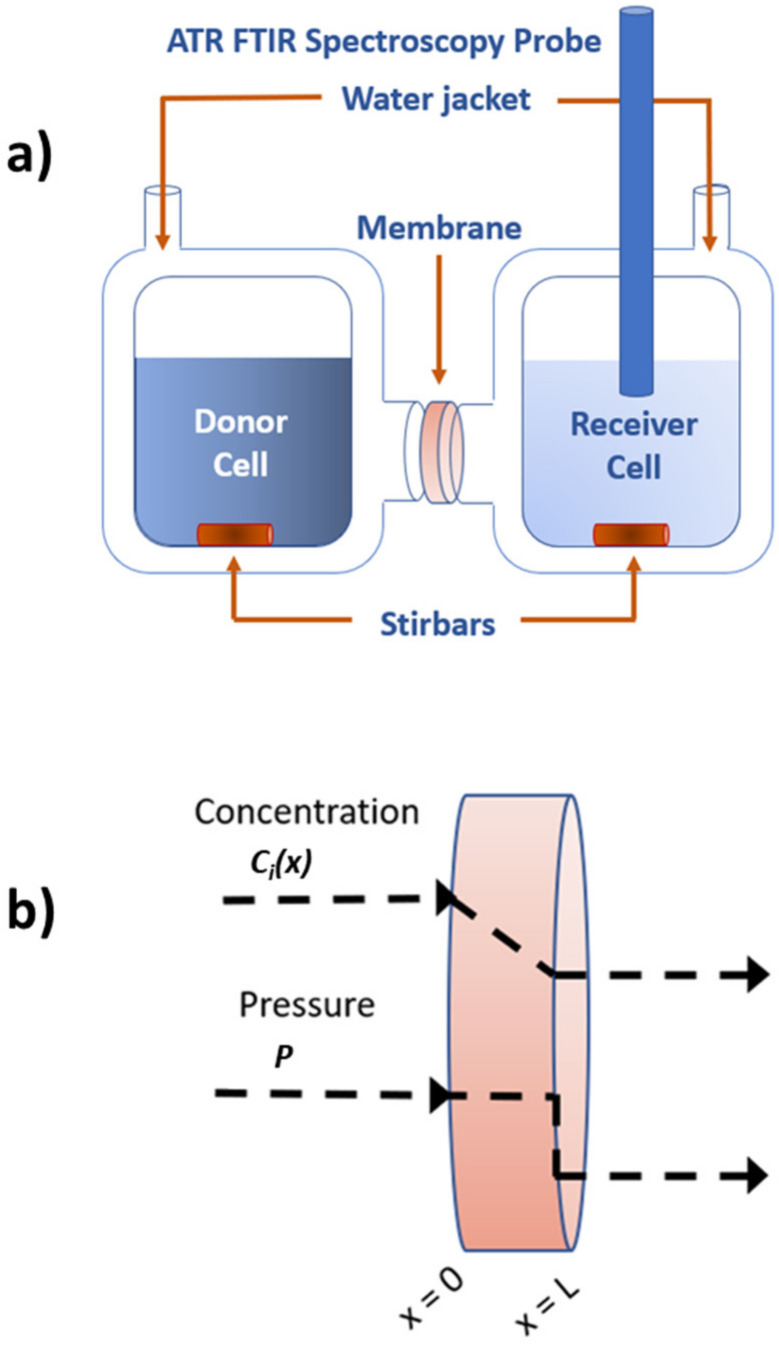
(**a**) Depiction of the diffusion cell apparatus using an in situ ATR FTIR spectroscopy probe (Section 3) and (**b**) concentration and pressure gradients throughout the membrane according to the solution-diffusion model.

The permeability, *P_i_*, can be measured experimentally using a diffusion cell apparatus (Figure 1a). A typical diffusion cell apparatus consists of a donor cell and receiver cell sandwiched around a hydrated membrane of known thickness. The receiver cell solution typically begins as pure deionized water (DI water) while the donor cell solution contains the solute(s) of interest in known concentration. Over time, the solute(s) diffuse across the membrane from the donor cell to the receiver cell down the concentration gradient(s) of the solute(s), shown in Figure 1b.

Typically, in situ probes such as pH [13,14] and conductivity [15,16,17,18] have been used to determine the change in solute concentration in the receiver cell over time. These measurements, however, cannot distinguish between different solutes, as they capture an overall solution property. Further, some solutes such as methanol do not change the pH or conductivity to any meaningful extent. Therefore, ex situ characterization techniques such as gas chromatography [19,20], mass spectrometry [21], and attenuated total reflectance Fourier transform infrared (ATR FTIR) spectroscopy [22] have been used to determine the change in concentration of multiple solutes over time. These characterization techniques, however, rely on the periodic acquisition of aliquots, increasing the probability of user error as well as changing the solution volume in the receiver cell. Recently, in situ ATR FTIR spectroscopy has allowed for continuous monitoring of multiple solutes without the need for aliquotic sampling. Beckingham et al. [23] showed that using in situ ATR FTIR spectroscopy to extract receiver cell concentrations yielded a permeability of methanol in the commercial cation exchange membrane Nafion^®^ that was similar to that reported elsewhere using other techniques, while also uncovering previously unreported differences between single-solute and multicomponent transport for mixtures of methanol and two carboxylates (sodium acetate and sodium formate). Dobyns et al. [12] used in situ ATR FTIR spectroscopy to study diffusion of multicomponent solutions of methanol and sodium acetate in membranes made from poly(ethylene glycol) diacrylate (PEGDA). In that work, the permeability to methanol increased in the presence of sodium acetate as a co-solute while the permeability to sodium acetate decreased, compared to single-solute measurements. Subsequently, Kim et al. [24] incorporated 2-acrylamido-2-methylpropanesulfonic acid (AMPS) as a comonomer with PEGDA to fabricate crosslinked cation exchange membranes and again found differences between single-solute and multicomponent permeabilities of methanol and sodium acetate. These effects are not restricted to alcohol/carboxylate solutions; for example, differences between single-solute and multicomponent permeabilities have also been observed for solutions of different alcohols. Dobyns et al. [25] investigated the permeabilities of solutions containing up to four solutes (methanol, ethanol, *n*-propanol, and acetone) for the commercial cation exchange membrane Nafion^®^ 117. The membrane selectivity to an aqueous solution of ethanol and *n*-propanol varied by up to 60% compared to single-solute measurements.

This prior work has demonstrated the importance of investigating multicomponent transport behavior in hydrated, dense polymer membranes. However, much remains unknown, motivating additional investigation. The following provides a review of the key considerations for the modeling (Section 2) and the experimental investigation (Section 3) of multicomponent diffusion. Both are needed to understand the behavior of these complex systems (Section 4).

## 2. Modeling of Multicomponent Diffusion

In this section, we review models for multicomponent diffusion using the framework of the solution-diffusion model because it is commonly applied to interpret experiments of diffusive transport across dense, hydrated membranes. Other modeling approaches, such as the pore-flow model or the recently proposed fluid–solid model [26], are considered outside the scope of this review.

### 2.1. Solution-Diffusion Model

In the solution-diffusion model [27,28,29], the $n=2+n_{s}$ component system comprising the membrane, the solvent, and *n*_s_ solutes is treated as a single thermodynamic phase. The pressure within this phase is constant and equal to that of the feed solution; however, the permeate solution can be at a lower pressure, e.g., in reverse osmosis processes. This pressure drop—modeled as a step change at the permeate boundary (Figure 1b)—must be mechanically supported, for example, by a porous layer. Because the pressure is constant within the membrane, transport of solutes is driven by diffusion due to concentration gradients. Typically, the solute composition in the feed is prescribed, and the composition of the permeate solution is determined by a combination of thermodynamic and transport considerations that determine the membrane’s performance.

The solvent and solute concentrations inside the membrane at the feed and permeate boundaries are assumed to be at chemical equilibrium with the solutions they contact, i.e., for the solvent and solutes to have equal chemical potentials inside and outside the membrane boundaries. This equilibrium can be characterized by the solubility, $K_{i}=c_{i}\left(0\right)/c_{i,f}$, where $c_{i}\left(x\right)$ is the concentration (number density) profile of component *i* inside the membrane with thickness *L* oriented along the *x*-axis (feed boundary x=0), and *c_i_*_,f_ is the concentration of component *i* in the feed solution (Figure 1b). Note that *K_i_* is a thermodynamic property that generally depends on composition, both in the membrane and in the feed solution, but is frequently assumed to be a constant for a given solute–membrane pair. However, in multicomponent solutions, *K_i_* may increase or decrease due to the presence of other solutes that enhance or reduce solubility of component *i*, respectively, in ways that are challenging to anticipate.

After adsorbing into the membrane, solutes also transport at different rates due to differences in their interactions with the membrane. The total number flux of component *i* is $c_{i}v_{i}$, where *v_i_* is the average velocity of component *i*. The diffusive flux $j_{i}=c_{i}\left(v_{i}-v\right)$ is defined as the flux relative to advection at a reference velocity *v*. Different reference velocities can be useful for analysis in different situations. For example, a common choice of *v* for bulk solutions is the barycentric (mass-averaged) velocity, $v=\sum_{i}\omega_{i}v_{i}$ where $\omega_{i}$ is the mass fraction of component *i*, but the mole-averaged velocity (using the mole fraction *x_i_* of component *i* instead of $\omega_{i}$) and the volume-averaged velocity (using the volume fraction $\phi_{i}$ of component *i* instead of $\omega_{i}$) are also used. For membranes, it can be particularly helpful to define $v=v_{m}$, where *v_m_* is the velocity of the membrane itself, because the membrane is stationary in the laboratory frame ($v_{m}=0$) while the barycentric velocity may be nonzero and unknown.

The diffusive flux, *j_i_*, can be modeled using several approaches, each parametrized by different types of diffusion coefficients. Care must be taken when applying these models because some diffusion coefficients depend on the reference velocity. A simple model for *j_i_* is Fick’s law, which when defined relative to the barycentric velocity is [30]

(2) $$j_{i}=-\frac{\rho D_{i}}{M_{i}}\frac{\partial \omega_{i}}{\partial x}\;,$$

where $\rho =\sum_{i}M_{i}c_{i}$ is the total mass density, *M_i_* is the molecular weight of component *i*, and *D_i_* is the mutual (Fick) diffusion coefficient of component *i*. If ρ is independent of composition, the familiar expression $j_{i}=-D_{i}\partial c_{i}/\partial x$ is obtained.

In diffusion-cell experiments (such as those described in Section 1), the solution-diffusion model with Fick’s law is commonly applied to extract *D_i_*. Assuming both ρ and *D_i_* are independent of composition, the steady-state flux across the membrane is

(3) $$j_{i}=D_{i}\frac{c_{i}\left(0\right)-c_{i}\left(L\right)}{L}=P_{i}\frac{c_{i,f}-c_{i,p}}{L}\;,$$

where *c_i_*_,p_ is the concentration of component *i* in the permeate solution, and we have assumed the same solubility, *K_i_*, on the feed and permeate boundaries. The permeability, *P_i_*, of component *i* can be obtained from experimental measurements of *c_i_*_,p_ in the receiver cell. For example, the commonly used Yasuda model, which can be obtained from Equation (3) [31], is

(4) $$\ln\left(1-2\frac{c_{i,p}\left(t\right)}{c_{i,p}\left(0\right)}\right)=-2\frac{P_{i}A}{LV_{p}}t\;,$$

where $c_{i,p}\left(t\right)$ is the concentration of component *i* in the permeate solution (receiver cell) at time *t*, *A* is the area of the membrane, and *V_p_* is the volume of solution in the receiver cell. The Fick diffusion coefficient, *D_i_*, can then be extracted from *P_i_* if the solubility, *K_i_*, is known.

When applied to multicomponent solutions, *P_i_* and *D_i_* extracted in this way can show complex dependencies on composition that are challenging to anticipate or interpret (Section 1). These dependencies need not be the same for both *P_i_* and *D_i_*, as *P_i_* includes the composition dependence of *K_i_* but *D_i_* does not. Moreover, *D_i_* is implicitly a pseudobinary diffusion coefficient associated with another dominant component such as the membrane and using this model for a multicomponent solution effectively neglects coupled transport due to solute–solute interactions.

### 2.2. Multicomponent Diffusion

The framework of nonequilibrium thermodynamics [32,33,34] gives a systematic approach to model multicomponent diffusion effects that are not captured by Equation (2). The diffusive flux, absent temperature or pressure gradients, is assumed to be proportional to gradients of the chemical potentials of all components,

(5) $$j_{i}=\overset{n}{\underset{k=1}{\sum }}L_{ik}\frac{\partial }{\partial x}\left(-\frac{\mu_{k}}{T}\right)\;,$$

where *L_ik_* is the Onsager coefficient coupling the flux of component *i* to the gradient of the chemical potential, $\mu_{k}$, of component *k* and *T* is the temperature. Not all *L_ik_* are independent: the Onsager coefficients can be written as a matrix that must be symmetric and positive semi-definite as consequences of microscopic reversibility and the second law of thermodynamics. Further, the diffusive fluxes are defined relative to the reference velocity *v* so only n−1 of the fluxes *j_i_* are independent, e.g., for the barycentric reference velocity, $\sum_{i=1}^{n}M_{i}\;j_{i}=0$. Simultaneously, the chemical potential gradients are constrained by the Gibbs–Duhem relationship (assuming local thermodynamic equilibrium),

(6) $$\overset{n}{\underset{i=1}{\sum }}c_{i}\frac{\partial \mu_{i}}{\partial x}=0$$

at constant temperature and pressure, so only n−1 of the chemical potential gradients are independent. These equations imply additional relationships between the $n^{2}$ Onsager coefficients, and only a subset must actually be determined [32,33,34,35]. Note that the Onsager coefficient *L_ik_* does not have typical dimensions of a diffusion coefficient; an Onsager diffusion coefficient, $\Lambda_{ik}=L_{ik}k_{B}/c$ where $c=\sum_{i=1}^{n}c_{i}$ is the total concentration, is sometimes defined [36]. The Onsager coefficients are also phenomenological and depend on the reference velocity so, as a result, can be challenging to interpret.

The Maxwell–Stefan approach is a popular alternative to Equation (5) with a simpler-to-interpret physical basis [37]. The chemical potential gradient on each component is thought of as a driving force that is balanced by friction forces with the other components. This picture leads to an implicit definition of the diffusive fluxes,

(7) $$-\frac{1}{k_{B}T}\frac{\partial \mu_{i}}{\partial x}=\overset{n}{\underset{\begin{array}{c} k=1\\ k\neq i \end{array}}{\sum }}{Đ}_{ik}^{-1}x_{k}\left(\frac{j_{i}}{c_{i}}-\frac{j_{k}}{c_{k}}\right)\;$$

where *k*_B_ is the Boltzmann constant. The Maxwell–Stefan diffusion coefficients, *Đ_ik_*, are independent of the reference velocity, and, with some algebra, can be related directly to the Onsager coefficients [36,37,38]. Both Equations (5) and (7) use the chemical potential gradient as a driving force for diffusion, but these are not directly measurable in experiments and must be estimated from composition gradients using an activity-coefficient model. This makes the Onsager and Maxwell–Stefan diffusion coefficients challenging to determine.

A multicomponent generalization of Equation (2), written using composition gradients directly, is hence desirable. One way to express the multicomponent Fick’s law is [39]

(8) $$j_{i}=-\frac{\rho }{M_{i}}\sum_{k=1}^{n-1}D_{ik}\frac{\partial \omega_{k}}{\partial x}\;.$$

Here, one of the *n* components has been eliminated based on the dependence of one of the *n* fluxes and *n* mass fractions on the others. The multicomponent Fick diffusion coefficients, *D_ik_*, depend on the reference velocity. Equation (8) can be shown to systematically follow from Equations (5) and (7). A consequence of this is that the multicomponent Fick diffusion coefficients, *D_ik_*, can be decomposed into a dynamic contribution, related to the Onsager or Maxwell–Stefan diffusion coefficients, and a thermodynamic contribution, based on an activity-coefficient model for the chemical potentials.

### 2.3. Simulating Multicomponent Diffusion Coefficients

Regardless of the modeling approach, multicomponent transport coefficients are challenging to predict and to measure in experiments. The primary difficulties are that multiple diffusion coefficients, which are chemistry and composition dependent, must be determined, and all of them can in principle contribute to the measurable flux of a given component. Molecular dynamics simulations give a powerful route to determine multicomponent diffusion coefficients computationally [40].

Simulating gradient diffusion in a one-to-one analog to diffusion cell experiments is challenging because the length scales that can be modeled with current computing resources are significantly smaller than in experiments, so (1) interfacial effects can be overemphasized in simulations and (2) larger composition differences are typically needed in simulations to obtain a reasonable flux. Interfacial effects can be eliminated by simulating a bulk material (without a composition gradient) using periodic boundary conditions [41]. Here, diffusion coefficients must be determined using either nonequilibrium simulation techniques that impose artificial forces on molecules to create flux or equilibrium simulation techniques that passively observe molecular displacements and compute diffusion coefficients using fluctuation–dissipation relations [40]. With nonequilibrium techniques, care must still be taken that the imposed force is not too large, and multiple simulations may be needed to determine all diffusion coefficients. We will accordingly focus here on the equilibrium technique for its relative simplicity and because a single simulation can be used to extract all diffusion coefficients.

An equilibrium molecular dynamics simulation is first performed, and a trajectory of the positions and velocities of all molecules is recorded at regular time intervals. The Onsager coefficients *L_ik_* can then be determined by analyzing the trajectory using [36,37,38,39,40]

(9) $$L_{ik}=\underset{t\to \infty }{\lim}\frac{Vc_{i}c_{k}}{6\;k_{B}}\frac{d}{dt}<\Delta r_{i}\left(t\right)\cdot \Delta r_{k}\left(t\right)>\;,\;$$

where *V* is the simulation volume, $\Delta r_{i}\left(t\right)$ is the displacement of the average position of the molecules of component *i* at time *t*, and angle brackets denote an ensemble average. Multiple time origins can be used within the trajectory to improve statistics. This equation is sometimes written in an equivalent form using a factor of 1/*t* rather than the time derivative [41]. A related equation based on velocity autocorrelation functions can also be used [38].

In standard molecular dynamics simulations, the barycentric velocity is zero because Newton’s equations of motion conserve linear momentum. Accordingly, it may be necessary to convert the measured Onsager coefficients if another reference velocity, e.g., where the velocity of the membrane is zero, is needed. Once *L_ik_* are determined, the Maxwell–Stefan diffusion coefficients can be directly computed. The multicomponent Fick diffusion coefficients require an additional thermodynamic model.

The described theoretical models are summarized in Table 1 for easy comparison.

## 3. Experimental Approaches to Investigating Multicomponent Diffusion

The permeability of a solute through a hydrated, dense membrane is a material-specific property that depends on the chemistry/structure of the membrane, the solute(s), and the solvent [31]. Within the solution-diffusion model using Fick’s law (Section 2.1), the permeability, *P_i_*, is modeled as a simple product of the solubility, *K_i_*, and diffusivity, *D_i_* (Equation (1)). Thus, a common experimental approach for determining the diffusivity of the solutes is to (1) measure the permeabilities of the solutes through the membrane (e.g., via a diffusion cell experiment), (2) measure the solubility of the solutes through the membrane via an independent sorption–desorption experiment, and then (3) calculate the diffusivities. Here, we examine the experiments used to determine the permeability and solubility of a solute in a hydrated, dense polymer membrane.

Historically, multiple experimental apparatuses have been used to measure the diffusion coefficients of more than one solute in a solution. In particular, interferometry was a widely practiced technique for performing transport studies of liquids [42,43]. This technique is based on observing the interference pattern that results from constructive and destructive interference of electromagnetic (light) waves, which provides information about the refractive index variation in the liquid layers adjacent to membranes [42]. There are multiple apparatuses for multicomponent interferometric diffusion studies, all having the general advantages that they are non-invasive, highly accurate, and do not require calibration. Robinson [44] and Crank and Robinson [45] studied the diffusion of chloroform, acetone, and methyl alcohol into a cellulose acetate membrane utilizing a Fabry–Pérot interferometer. In this study, the membrane was sandwiched between two partially reflecting glass plates and the penetration depth of the liquid into the membrane was measured as a function of time [42,44]. Spiegler et al. [46] analyzed concentration polarization effects in electrodialysis cells using both microelectrodes and a Fabry–Pérot interferometer. Later, Lerche [47] utilized the Mach–Zehnder interferometer for studying the transport of aqueous sodium chloride across a cellulose membrane at atmospheric pressure. Other transport studies were performed by Johnson [48] using a Mach–Zehnder interferometer, Bollenbeck [49] using a Raleigh interferometer to study liquid phase diffusion of aqueous solution through a semi-permeable membrane, and Welinder [50] using holographic interferometry to observe concentration profile within an electrodialysis membrane. However, a disadvantage of measuring multicomponent diffusion using interferometry is that the approach can be quite cumbersome, involving complicated setups, time-consuming experiments, and, to some extent, arduous data analysis [43].

By contrast, a diffusion cell apparatus (Figure 1) is simpler to construct and directly measures permeability. This has led many prior techniques to fall out of favor and given way to diffusion cells becoming a dominant methodology for investigating diffusive transport in membranes. To measure single-solute or multicomponent permeability using a diffusion cell apparatus, a membrane of known thickness is sandwiched between donor and receiver cells with a known orifice area between them. Solutes permeate from the high concentration donor cell to the low concentration receiver cell through the membrane.

To determine the concentration of a single-solute in the receiver cell solution, a pH meter or a conductivity meter is often suitable; a change in solution pH or conductivity is proportional to the solute concentration. These methods are relatively inexpensive and convenient as modern meters allow for continuous data logging. An important assumption of the solution-diffusion model is the concentration of the solute in bulk solutions and near the membrane interface are the same [28,29]. Since measuring the concentration at the membrane–solution interface in diffusion cell experiments is quite challenging, this assumption helps to experimentally determine the solute concentration at the bulk phase. Therefore, solute concentration is typically measured in the bulk phase through analytical techniques. However, other techniques are needed to extract the permeability of individual solutes in multicomponent solutions because neither pH nor conductivity can discriminate between specific chemical moieties within a mixture.

Spectroscopic techniques are good alternatives to measure the concentration of each of the solutes in multicomponent solutions. Gas chromatography [19,20,51], ion exchange chromatography [52,53], flame atomic adsorption spectroscopy [54], high-performance liquid chromatography (HPLC) [55,56], and UV/Vis spectroscopy [57] all are ex situ techniques that might be used to analyze samples containing different solutes. For example, Varcoe et al. [58] investigated the permeability of a polymer electrolyte membranes to methanol, ethanol, and ethylene glycol. They evaluated the alcohols’ permeabilities by monitoring the concentration over time using gas chromatography with a flame ionization detector and SolGel-WAX capillary column. As another example, McCormick et al. [56] investigated water, salt, and ethanol diffusion in a forward osmosis process. In this study, a diffusion cell setup was used, and the concentrations of ethanol and salt were analyzed using HPLC. Although they can discriminate between solutes, ex situ spectroscopic quantification methods have the main disadvantage that they require aliquotic sampling from the receiver chamber. Aliquotic sampling can not only be time-consuming but also complicate analysis. The Yasuda model (Equation (4)) assumes the total receiver-cell volume is constant but aliquotic sampling removes liquid. The removal of aliquots may also create a hydrostatic pressure difference between the cells that can lead to advective flow. Hence, ex situ methods are less appealing for extracting permeability using the Yasuda model.

**Figure 2:**
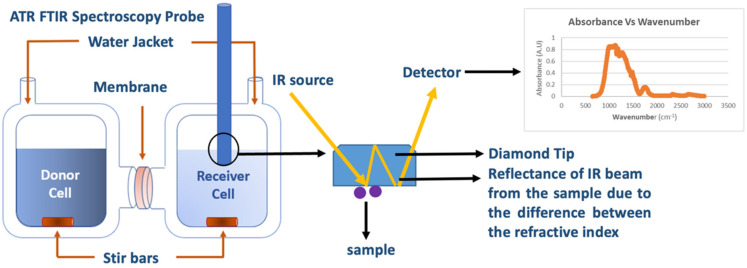
Schematic of multicomponent permeability measurement through a polymeric membrane using a diffusion cell coupled with in situ ATR-FTIR spectroscopy.

Fortunately, a facile in situ spectroscopy technique using ATR FTIR spectroscopy was introduced by Carter et al. [59]. The technique measures the infrared absorbance in the receiver cell allowing for the extraction of solute concentrations for mixtures as shown in Figure 2. Prior to this adaptation for diffusion cells, ATR FTIR spectroscopy was utilized to perform transport studies in polymer films [60,61,62,63,64,65]. For example, Wu et al. [61] used ATR FTIR spectroscopy to study the water diffusion into an epoxy resin, while Doppers et al. [62] used it to observe the diffusion of water and acetone into poly(vinyl alcohol)–clay nanocomposites. Elabd [63] and Hallinan [64] and measured the permeability of methanol through Nafion^®^ 117 by circulating the receiver cell solution through a benchtop ATR FTIR spectrometer. A later study by Carter et al. [59] on multicomponent transport of alcohols through Selemion AMV removed the need for recirculation, along with the accompanying time-delay and leakage issues.

The working principle for the in situ ATR FTIR spectroscopy method relies on the fact that every organic molecule shows a distinct absorbance peak in a particular wavelength due to the stretching or bending of the bonds between the atoms within the molecule. Due to the difference between the refractive indices between each of the molecules and the diamond tip, IR radiation reflects and gets absorbed based on the bending/stretching energy at the diamond tip/solute interface multiple times. This technique is thereby ideal for monitoring the concentrations of molecules with detectable bond signatures (organic molecules and organic salts), but it is not capable of detecting inorganic salts (e.g., NaCl) that lack detectable IR signatures.

The absorbance spectra of each solute can be translated into concentration data using the Beer–Lambert law:

(10) $$A_{\lambda }=\log\left(\frac{I_{0}}{I}\right)=E_{\lambda }lc\;,$$

where $A_{\lambda }$ and $E_{\lambda }$ are the absorbance and molar absorptivity of the solute at the wavenumber λ, *I* and *I_0_* are the transmitted and incident intensity of light, and *l* is the incident light’s path length which travels through the solution. This relationship can be written compactly by defining the effective molar absorptivity $\epsilon_{\lambda }=E_{\lambda }l$. If the solution contains multiple species, the Beer–Lambert law is additive,

(11) $$A_{\lambda }=\overset{n_{s}+1}{\underset{i=1}{\sum }}\epsilon_{\lambda ,i}c_{i},$$

where $\varepsilon_{\lambda ,i}$ is the effective molar absorptivity of solute *i* at λ, $c_{i}$ is the concentration of solute *i* of interest, and *n_s_* is the number of solutes. Both the solutes and solvent are included in this summation (*n_s_*+1) as the general case since the concentration of solvent changes corresponding to the concentration of solutes. However, this is usually accounted for through background subtraction of the receiver cell absorbance at *t* = 0 and by choosing wavenumbers for the solutes that are unaffected by the solvent spectra.

**Figure 3:**
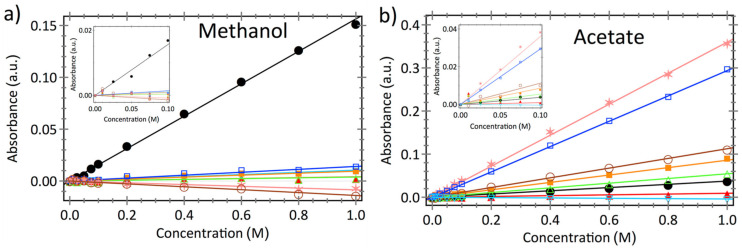
Absorbance data obtained from in situ ATR FTIR plotted against various concentrations in ultrapure water for (**a**) methanol, (**b**) sodium acetate, at various wavenumbers showing desired linear relationship. Reprinted (adapted) with permission [23]. Copyright 2022 Elsevier.

To apply Equation (11), it is necessary to know the effective molar absorptivity $\epsilon_{\lambda ,i}$ for each solute of interest. For calibrating the equipment and to get the molar absorptivity, the following procedure is usually followed. At first, a series of aqueous solutions with varying concentrations (0.025–0.25 M) of solutes of interest are prepared and their spectra recorded. A wavenumber λ is selected for each of the solutes where a distinctive peak is observed. In the study by Beckingham et al. [23], methanol, sodium formate, and sodium acetate were investigated, and their absorbance was measured at several wavenumbers for a range of concentrations. The effective molar absorptivity of each solute at each chosen wavenumber was extracted by linear least-squares regression fit. Among several wavenumbers, the wavenumber where concentration data exhibited both high effective molar absorptivities and high correlation coefficients was chosen (1018 cm^−1^ for methanol, 1414 cm^−1^ for sodium acetate, and 1350 cm^−1^ for sodium formate) [23]. Figure 3 shows an example of the calibration process for methanol and sodium acetate. Once the molar absorptivity for each of the solute at a certain wavenumber are obtained, they are utilized to determine the concentrations from absorbance spectra. Time-resolved concentration data then yields the permeability of the membrane to each solute.

**Figure 4:**
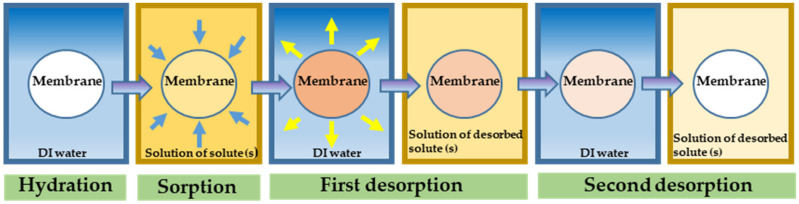
Schematic diagram of sorption-desorption experiment. (Left to right) Equilibration in DI water, Equilibration with solution of solute (s) of interest, followed by successive equilibrium desorption in DI water with solution concentrations determined by HPLC.

The second step to determine the diffusivity is to measure the solubility, *K*_i_, by performing a sorption–desorption experiment, shown schematically in Figure 4. Briefly, these experiments involve equilibrating the membrane with a donor solution and subsequently desorbing in order to determine how much of the solute(s) were taken up by the membrane by measuring the concentration of the solute(s) in the desorption solution using an appropriate method (HPLC, conductivity, etc.) The solubility, *K_i_*, is then calculated as the ratio of the concentration of solute in the membrane and the concentration of the donor solution. This allows for the diffusivity, *D_i_*, to then be calculated from the solution-diffusion model (Equation (1)).

Using in situ ATR FTIR spectroscopy for permeability and sorption-desorption experiment for solubility is an attractive approach for investigating relationships between membrane chemistry, solute mixtures, and transport behavior [12,23,24,25,66,67]. Dobyns et al. [12] investigated transport of methanol and sodium acetate in crosslinked PEGDA membranes, where differences in permeabilities to methanol and sodium acetate in co-transport relative to single solute transport were observed depending on the amount of fractional free volume in the membrane. A subsequent study by Kim et al. [24] incorporated AMPS, a sulfonated charged moiety, into the PEGDA-based membranes, and the permeability to sodium acetate increased while copermeating with methanol compared to single-solute permeation. A subsequent study by Kim et al. [66], found that by incorporating uncharged comonomers of different side-chain length—acrylic acid, hydroxyethyl methacrylate, and poly(ethylene glycol) methacrylate—where this behavior could be manipulated and ultimately that carboxylate crossover could be significantly suppressed in PEGMA containing films.

The described experimental methods are summarized in Table 2 for easy comparison.

## 4. Future Outlook and Conclusions

We have discussed a variety of theoretical (Section 2) and experimental (Section 3) considerations for characterizing diffusive transport through hydrated, dense polymer membranes. We note that multicomponent effects have typically been ignored in discussions of both the theory and experimental investigations of multicomponent transport for hydrated, dense polymer membranes as the additional complications have historically required complex analyses. Multicomponent transport behavior can be complex when there are multiple solutes simultaneously diffusing, with the membrane’s permeability to each solute depending on the combination of the membrane and solute chemistries and their array of interactions. Multicomponent diffusion in these types of membranes can be modeled and characterized by diffusion coefficients expressed using different formalisms (Fick, Onsager, and Maxwell–Stefan). These diffusion coefficients can also be simulated and extracted using molecular modeling approaches. However, experimentally measured permeabilities extracted from standard diffusion cell experiments do not provide much insight into the underlying multicomponent diffusion coefficients. Because the permeability is typically extracted from each individual solute’s concentration over time, only an effective mutual Fick diffusivity is usually recovered and information about possible coupled transport mechanisms is lost. In that way, experimental approaches do allow for investigations of differences in membrane transport at the permeability and solubility level, but modeling approaches are required for extracting additional insights. Conversely, without the experimental capability to easily measure permeability from multicomponent diffusion cell experiments or solubility from sorption–desorption experiments, the ability to parametrize and validate modeling approaches is hindered. With the new approach utilizing in situ ATR FTIR spectroscopy for measuring multicomponent receiver-cell concentrations, robust computational and experimental capabilities now exist to advance our understanding of multicomponent membrane transport. We anticipate joint application of both approaches will give rise to a tremendous opportunity to advance membrane science and design.

## Figures and Tables

**Table 1:** Summary of Discussed Theoretical Transport Models.

Model	Driving Force	Transport Coefficient	Notes
Solution-diffusion model with Fick’s law (Equation (3))	Composition gradient	*P_i_*	Based on Fick’s law for diffusion (Equation (2)), which neglects off-diagonal (*i* ≠ *j*) fluxes in Equation (8). *P_i_* is the product of the diffusivity, *D_i_*, and the solubility, *K_i_*.
Multicomponent Fick’s law (Equation (8))	Composition gradient	*D_ij_*	*D_ij_* can be related to *L_ij_* or *Đ_ij_* using a thermodynamic model.
Nonequilibrium thermodynamics (Equation (5))	Chemical potential gradient	*L_ij_* or $\Lambda_{ij}$	*L_ij_* are measurable in equilibrium molecular dynamics simulations (Equation (9)).
Maxwell–Stefan (Equation (7))	Chemical potential gradient	*Đ_ij_*	*Đ_ij_* are independent of reference velocity, can be computed from *L_ij_*.

**Table 2:** Summary of Discussed Experimental Approaches.

Experimental Approach	Variables to Measure	Significance
Interferometry	Measures refractive index variation in liquid layers adjacent to membrane	Measured refractive index used to calculate diffusivity coefficients (*D_i_*). Experiments and calculations are quite complex.
Diffusion-Cell experiments with aliquotic sampling	Measures solute(s) concentration(s) in receiver cell utilizing ex situ spectroscopic methods	Measured solute(s) concentration(s) used in Yasuda model to determine permeability (*P_i_*). For multicomponent systems sampling results in non- constant volume in cell and is aliquot analysis time-consuming.
Diffusion-Cell experiments coupled with in situ ATR FTIR	Measures solute (s) concentration(s) variation in receiver cell using in situ ATR FTIR spectroscopy.	Real time concentration data obtained from the diffusion cell for use in Yasuda’s model to extract multi-solute permeabilities (*P_i_*).
Sorption-desorption experiment	Measures concentration of solute desorbed from membrane after equilibrium sorption.	Desorbed solute(s) concentration(s) used with measured membrane volume to calculate membrane solubility (*K_i_*) to the solute(s).

## Data Availability

Not applicable.

## Nomenclature

*P_i_*	Permeability
*D_i_*	Fick’s law diffusivity
*K_i_*	Solubility
*n*	Number of components
*n_s_*	Number of solutes
*c_i_*	Concentration of component *i* inside the membrane
*c_i,f_*	Concentration of component *i* in feed solution
*c_i,p_*	Concentration of component *i* in permeate solution
*v_i_*	Average velocity of component *i*
*v*	Reference velocity
*j_i_*	Diffusive flux relative to reference velocity
ω _*i*_	Mass fraction of component *i*
*M_i_*	Molecular weight of component *i*
ρ	Total mass density
$\mu_{i}$	Chemical gradient of component *i*
*L_ij_*	Onsager coefficient
$\Lambda_{ij}$	Onsager diffusion coefficient
*Đ_ik_*	Maxwell Stefan diffusion coefficient
*D_ij_*	Multicomponent Fick diffusion coefficient
$A_{\lambda }$	Absorbance at wavenumber *λ*
$E_{\lambda }$	Molar absorptivity of the solute at wavenumber *λ*
*I*	Transmitted light intensity
*I_o_*	Incident light intensity
$\epsilon_{\lambda }$	Effective molar absorptivity
*l*	Light’s path length
